# Evaluating the Effectiveness of an Online Course on Pediatric Malnutrition for Syrian Health Professionals: Qualitative Delphi Study

**DOI:** 10.2196/53151

**Published:** 2024-10-28

**Authors:** Amal Sahyouni, Imad Zoukar, Mayssoon Dashash

**Affiliations:** 1 Medical Education Program, Syrian Virtual University, Al-Gomhoria Street, Lattakia, Syrian Arab Republic, 963 949391102; 2 Faculty of Medicine, Tishreen University, Lattakia, Syrian Arab Republic; 3 Pediatrics Department, Faculty of Medicine, Damascus University, Damascus, Syrian Arab Republic; 4 Faculty of Dental Medicine, Damascus University, Damascus, Syrian Arab Republic

**Keywords:** effectiveness, online course, pediatric, malnutrition, essential competencies, e-learning, health professional, Syria, pilot study, acquisition knowledge

## Abstract

**Background:**

There is a shortage of competent health professionals in managing malnutrition. Online education may be a practical and flexible approach to address this gap.

**Objective:**

This study aimed to identify essential competencies and assess the effectiveness of an online course on pediatric malnutrition in improving the knowledge of pediatricians and health professionals.

**Methods:**

A focus group (n=5) and Delphi technique (n=21 health professionals) were used to identify 68 essential competencies. An online course consisting of 4 educational modules in Microsoft PowerPoint (Microsoft Corp) slide form with visual aids (photos and videos) was designed and published on the Syrian Virtual University platform website using an asynchronous e-learning system. The course covered definition, classification, epidemiology, anthropometrics, treatment, and consequences. Participants (n=10) completed a pretest of 40 multiple-choice questions, accessed the course, completed a posttest after a specified period, and filled out a questionnaire to measure their attitude and assess their satisfaction.

**Results:**

A total of 68 essential competencies were identified, categorized into 3 domains: knowledge (24 competencies), skills (29 competencies), and attitudes (15 competencies). These competencies were further classified based on their focus area: etiology (10 competencies), assessment and diagnosis (21 competencies), and management (37 competencies). Further, 10 volunteers, consisting of 5 pediatricians and 5 health professionals, participated in this study over a 2-week period. A statistically significant increase in knowledge was observed among participants following completion of the online course (pretest mean 24.2, SD 6.1, and posttest mean 35.2, SD 3.3; *P*<.001). Pediatricians demonstrated higher pre- and posttest scores compared to other health care professionals (all *P* values were <.05). Prior malnutrition training within the past year positively impacted pretest scores (*P*=.03). Participants highly rated the course (mean satisfaction score >3.0 on a 5-point Likert scale), with 60% (6/10) favoring a blended learning approach.

**Conclusions:**

In total, 68 essential competencies are required for pediatricians to manage children who are malnourished. The online course effectively improved knowledge acquisition among health care professionals, with high participant satisfaction and approval of the e-learning environment.

## Introduction

Severe acute malnutrition (SAM) increases the risk of mortality among children aged younger than 5 years, affecting an estimated 17 million children worldwide, particularly in low- and middle-income countries [1,2]. The Syrian conflict has exacerbated this crisis, with half a million children enduring chronic malnutrition and 137,000 aged younger then 5 years experiencing acute malnutrition, increasing their susceptibility to preventable diseases [3]. Scaling up the management of SAM is crucial for reducing child mortality [4], but training and resourcing health care professionals to effectively identify and treat children who are malnourished remain major challenges, especially in conflict zones such as Syria [5,6].

While a wealth of knowledge exists about pediatric malnutrition, a crucial gap remains. This gap lies in the delivery of practical, accessible, and contextually relevant training for health care professionals in conflict zones [7]. This study addresses this gap by focusing on the development and evaluation of a self-directed online course for Syrian pediatricians and other health care professionals in the management of pediatric malnutrition.

e-Learning offers a promising solution for addressing this training gap, providing a flexible, scalable, and cost-effective method to deliver high-quality instruction [8]. By using e-learning, we can empower health care professionals with the necessary knowledge and skills to combat pediatric malnutrition; bridge access barriers, particularly in conflict zones; and tailor training to meet specific needs [7,9].

However, existing e-learning platforms may not adequately address the unique challenges and context of conflict zones such as Syria, where resources are limited [10]. Local training initiatives are crucial to ensure contextual relevance and maximize impact. By identifying specific knowledge gaps and skill deficits, developing culturally sensitive materials, and integrating with local resources, tailored e-learning solutions can foster a sense of ownership and engagement, leading to more effective knowledge transfer and application [11].

This study investigates the efficacy of e-learning as a training solution for pediatric malnutrition management in Syria, a context marked by conflict and limited access to traditional training opportunities. Specifically, this study aims to identify essential competencies needed by pediatricians and health care professionals for effective management of pediatric malnutrition, evaluate the efficacy of an online course on pediatric malnutrition in improving their knowledge and skills, and explore the potential of e-learning as a scalable solution for training in challenging contexts. By addressing this crucial training gap, the research seeks to contribute to improved patient care; enhanced staff retention; and, ultimately, a reduction in pediatric malnutrition in Syria.

## Methods

### Ethical Considerations

This study was approved by the Ethical Committee at the Syrian Virtual University (SVU; #2154/0, November 25, 2021). An electronic consent form was obtained from all participants, ensuring their understanding that their data would be kept confidential and used solely for the purposes of this research study.

### Competency Development

#### Overview

The Delphi technique was used to develop a consensus regarding essential competencies for managing children who are malnourished, as it is a helpful strategy for identifying medical education competencies [12]. Further, 2 qualitative methods were applied sequentially—focus groups and the Delphi technique.

#### Focus Groups

The online focus group consisted of 5 participants: 3 postgraduate students working in an inpatient stabilization center in Lattakia and 2 pediatricians serving as pediatric malnutrition therapists on projects funded by UNICEF (United Nations Children’s Fund). The participants were recruited via telephone and social media to participate in the virtual discussion.

The research approach was fully explained to the focus group participants, who were asked to provide a brief report on the competencies needed by pediatricians to manage pediatric malnutrition. Consequently, a preliminary list of essential competencies was formulated during a 3-hour meeting.

#### Delphi Rounds

All pediatricians and health professionals enrolled in the Medical Education Master Program at the SVU were invited to participate in this study (n=21). Ultimately, 18 of them took part. Participants were instructed, via the Virtual University Management System, to review relevant protocols and guidelines on pediatric malnutrition published by recognized health organizations, such as the World Health Organization (WHO) and UNICEF [13,14]. A methodology for writing competencies and vocabulary for job descriptions [15] was established to prepare the initial competency list.

Participants were randomly assigned to 1 of 3 groups, each focused on knowledge, skills, or attitudes, for the identification of essential competencies.

Through 3 virtual meetings conducted over a month, all groups agreed on a classification of competencies based on etiology, assessment, diagnosis, and managing pediatric malnutrition. The team leader reviewed all responses, removed repetitive items, reformulated inappropriate ones, and discussed transferring fields between specialties. Participants then independently reviewed the competencies over a 1-week period, and the revised list was distributed to all participants for rating on a 5-point Likert scale within a week.

Competencies endorsed by at least 80% (15/18, 83%) of participants were subsequently combined and merged. The weighted response for each competency was obtained by calculating the responses at each level and the mean score for each competency, ranging from 0.0 to 3.0 [16]. All competencies were ranked, and the relative importance of each competency was identified.

### Training Development

#### Course Design

Drawing upon a variety of sources and expertise, an online pediatric malnutrition course was developed, informed by the competencies identified in the first phase of this study. The course was designed to meet the specific needs and context of Syrian health professionals, taking into account the unique challenges and priorities of the Syrian health care system.

The development of the online course and its accompanying multiple-choice questions involved a collaborative effort among pediatricians possessing years of experience in managing both inpatients and outpatients with acute malnutrition, as well as in training health professionals.

Available evidence-based guidelines and protocols from reputable organizations, such as the WHO and UNICEF, were carefully reviewed to ensure the content validity and accuracy of the course materials. Visual aids, such as images and videos, were also incorporated to enhance engagement and learning.

#### Course Content

The e-learning content was categorized into 4 modules covering all identified competencies. Module 1 provided information on definition, classification, prevalence, differentiation between types, and associated pathophysiological changes in pediatric malnutrition. Module 2 focused on the diagnosis and anthropometric measurement techniques for accurate assessment. Module 3 presented treatment modalities according to WHO guidelines and the 10-steps approach. Module 4 addressed the prognosis and medical complications associated with pediatric malnutrition. Each module contained interlinked subunits for easy offline access, designed to be completed in less than 3 hours.

The principal investigator, AS, developed online content, incorporating visual aids such as images and videos, and created 40 multiple-choice pre- and posttests. The course was delivered in Arabic.

### Participants

#### Recruitment

This study’s participants comprised 10 individuals: 5 pediatricians and 5 other health care professionals, including medical doctors (general practitioners and specialists), dentists, and pharmacists. These participants, 8 females and 2 males, were recruited via social media to evaluate the online course.

Recruitment for this study was announced via the researcher’s email and on social networking sites within official groups for doctors and resident students. Participation was optional, and the purpose of the research was clearly communicated as solely for scientific purposes. Participant grades would not be used outside the research and would remain confidential. Participants provided explicit consent via email, with a “yes” or “no” response.

#### Training Delivery

The SVU’s learning management system was used for the online course presentation, with access restricted to participants via individual usernames and passwords. Data security was maintained through the platform’s security protocols.

Prior to accessing the course, participants were surveyed regarding their prior attendance in malnutrition-related training courses. A designated date for the pretest was communicated via email to each participant. Upon completion of the pretest, participants were granted access to the online course, with a 1-month time frame allotted for completion. Following course completion, a second email was sent to each participant, scheduling a specific date for the online, synchronous posttest.

### Evaluation

#### Assessment Instruments

Both the pretest and posttest, administered electronically via a Google Forms link within the SVU platform, consisted of 40 identical multiple-choice questions, ensuring a consistent measure of knowledge acquisition. The assessment instrument was distributed across the 4 modules, with a weighting reflecting the number of subunits within each module: modules 1 and 2, covering foundational knowledge of pediatric malnutrition, each contained 10 questions; module 3, focusing on assessment and diagnosis, included 5 questions; and module 4, on management, contained 15 questions.

#### Participant Satisfaction

Participant satisfaction was assessed using a 30-item questionnaire encompassing 3 domains: content presentation style, knowledge gained, and the e-learning environment (10 items per domain). Participants rated each item using a 5-point Likert scale ranging from 0 (strongly disagree) to 4 (strongly agree). Cronbach α, a measure of internal consistency [17], was calculated. The mean and SD were then used to analyze the distribution of responses for each item, providing insights into participant satisfaction levels across the different domains.

The qualitative interpretation of Likert scale measurements is shown in Table S1 in Multimedia Appendix 1.

#### Data Analysis

To evaluate knowledge gains, paired *t* tests (2-tailed) were used to compare pre- and posttest scores. Subgroup analyses were conducted to examine potential differences in knowledge acquisition based on participant characteristics, including gender, specialty (pediatricians or other health professionals), and prior training experience. Comparisons between groups (eg, male vs female) were facilitated using the Mann-Whitney test, while comparisons within groups (eg, pretest vs posttest scores for pediatricians) were performed using the Wilcoxon signed rank test [18,19].

These nonparametric tests were chosen due to the relatively small sample size (n=10) [20], followed by parametric tests, and the results were compared. Statistical significance was set at *P*<.05.

IBM SPSS Statistics for Windows (version 25.0; IBM Corp) was used to perform all the analyses.

## Results

The Delphi technique yielded an 85% (18/21) response rate. At least 80% (15/18, 83%) of participants suggested 68 essential competencies for managing children who are malnourished, organized into knowledge, skills, and attitude. As outlined in Table 1, competencies also fall under 3 subheadings: etiology, assessment or diagnosis, and management.

**Table 1.** Essential competencies for managing children who are malnourished.

Domain		Competency
**Knowledge**		
	Etiology	Recognize malnutrition terminology. Recognize the epidemiology of malnutrition in children. Differentiate between types of malnutrition. Identify the causes and prevalence of pediatric malnutrition worldwide.
	Assessment and diagnosis	Identify the clinical signs and symptoms of acute malnutrition in children. Recognize different methods for assessing children who are malnourished. Describe admission and discharge criteria for managing SAM^a^ with medical complications under inpatient care. Identify high-risk groups for SAM in children. Discuss strategies to detect cases of pediatric malnutrition. Recognize complications and prognosis of pediatric malnutrition. Identify target age groups to screen for malnutrition. Explain methods for diagnosing malnutrition in children.
	Management	Describe outpatient care for managing SAM without medical complications. Describe admission criteria for outpatient care (infants under 6 mo and children 6‐59 mo) Describe the outpatient care and follow-up process for children 6‐59 mo. Explain medical treatment for SAM without complications under outpatient care. Explain nutrition rehabilitation for SAM without complications under outpatient care for children 6‐59 mo. Describe the key messages for mothers or caregivers of children 6‐59 mo in outpatient care. Explain managing at-risk mothers and infants aged younger than 6 mo without complications in outpatient care. Explain discharge criteria and procedures for at-risk mothers, infants under 6 mo, and children 6‐59 mo. Outline management of SAM with medical complications under inpatient care. Review medical and dietary treatment in inpatient care. Describe programs to manage MAM^b^. Describe the admission and discharge process for MAM management.
**Skills**		
	Etiology	Classify nutritional vulnerability in at-risk mothers and infants aged younger than 6 mo. Educate parents of children who are malnourished to understand the risks of pediatric malnutrition. Develop a plan to monitor cases of pediatric malnutrition. Monitor and respond to barriers to access.
	Assessment and diagnosis	Take accurate clinical history. Measure the weight, length, and mid-upper arm circumference of children. Calculate the child’s age. Classify acute malnutrition. Perform appropriate medical examination. Provide correct diagnosis in each pediatric malnutrition case. Assess and admit a child to outpatient care. Assess and manage at-risk mothers and infants under 6 mo without medical complications in outpatient setting. Conduct field visits for children who are malnourished in supplementary feeding programs.
	Management	Provide outpatient care for SAM without medical complications. Identify when further action is required, such as referral to inpatient care and follow-up home visits. Treat a child during outpatient care follow-on. Practice referral between inpatient care and outpatient care. Make referrals from supplementary feeding to outpatient or inpatient care. Complete patient records and interpret findings. Calculate and review service or program performance. Calculate therapeutic doses accurately. Correctly apply treatment in terms of timing and adjustments for each case. Accurate diagnosis of pediatric malnutrition. Manage clinical cases based on stage, development, and complications. Discuss medical and nutritional treatment for MAM management. Discuss treatment protocols according to malnutrition severity in a child. Apply therapeutic protocols. Administer therapeutic foods according to the malnutrition severity. List indications and contraindications of medications and procedures.
**Attitude**		
	Etiology	Explain all malnutrition information to parents. Promote health education about malnutrition and when to take action.
	Assessment and diagnosis	Keep children who are malnourished safe and protected from harm. Demonstrate investigative and analytical thinking to meet the needs of children who are malnourished. Provide the best possible health care to children who are malnourished regardless of age, gender, culture, and economic status. Communicate effectively with children who are malnourished and families to explain case progression.
	Management	Demonstrate professionalism with peers, staff, patients, and families. Collaborate with health care professionals. Respect patient privacy and confidentiality. Show sympathy and compassion for children who are malnourished. Provide spiritual support to children who are malnourished and parents. Develop strategies for consultation, collaboration, and referral. Exhibit leadership, initiative, and optimism to manage cases effectively. Work flexibly under stress and changing conditions while remaining calm. Apply WHO^c^ general principles for routine care (10-steps).

a SAM: severe acute malnutrition.

b MAM: moderate acute malnutrition.

c WHO: World Health Organization

The final competencies comprised 24 knowledge, 29 skills, and 15 attitudes items. By competencies classification, 10 addressed etiology, 21 assessment or diagnosis, and 37 management.

In total, 10 participants were recruited between July 1, 2021, and July 15, 2021. Following the administration of the pretest, the online course commenced on July 21 and continued for 1 month. The posttest was administered on August 28, 2021, upon completion of the course. The cohort comprised 5 (50%) pediatricians and 5 (50%) other health professionals, with 8 (80%) females and 2 (20%) males. All pediatricians had prior experience in managing children who are malnourished and had received training on pediatric malnutrition, with 3 having been trained over a year prior and 2 within the past year. None of the other health professionals in the cohort had previous training.

Table 2 displays knowledge gained across participants, assessed before and after the course. Comparisons were made between groups based on gender, specialty, and prior training. All participants achieved higher posttest than pretest scores. The overall mean increase in knowledge scores from pretest to posttest was 11.0 points, representing a 45% relative gain. This significant difference (*P*<.001) indicates that the online course was effective in enhancing knowledge about pediatric malnutrition for the entire cohort.

**Table 2.** Knowledge gains between pre- and posttests based on participant demographics.

Variable	n	Pretest mean^a^(SD)	Posttest mean (SD)^a^	Mann-Whitney (*P* value)		Paired *t* test (*df*; *P* value)	
				Pretest	Posttest	Pretest	Posttest
Female	8	23.6 (6.7)	35.4 (3.7)	−0.26 (.79)	−0.53 (.60)	−0.58 (8;.58)	0.32 (8;.76)
Male	2	26.5 (2.1)	34.5 (0.7)	−0.26 (.79)	−0.53 (.60)	−0.58 (8;.58)	0.32 (8;.76)
Health professionals	5	20.2 (5.9)	33 (2.5)	−2 (.046)	−2.11 (.04)	−2.73 (8;.03)	−2.81 (8;.02)
Pediatricians	5	28.2 (2.9)	37.4 (2.4)	−2 (.046)	−2.11 (.04)	−2.73 (8;.03)	−2.81 (8;.02)
Nonprior attendance	5	20.2 (5.9)	—^b^	−2 (.046)	—	−2.73 (8;.03)	—
Prior attendance	5	28.2 (2.9)	—	−2 (.046)	—	−2.73 (8;.03)	—
More than 1 year	3	26.3 (1.5)	—	—	—	−3.43 (8;.04)	—
Last year	2	31 (1.4)	—	—	—	−3.43 (8;.04)	—
Total	10	24.2 (6.1)	35.2 (3.3)	−2.81 (.01)	−2.81 (.01)	−7.79 (9;<.001)	−7.79 (9;<.001)

a Maximum score=40.

b Not applicable.

Notably, the paired *t* test revealed a statistically significant improvement in posttest scores for all participants (5/5) with prior malnutrition training (*P*=.03). This finding suggests that the course not only builds upon existing knowledge but also serves as a valuable refresher for those previously trained in this area (Table 2).

Female participants had average pretest scores of 23.6 (SD 6.7) and posttest scores of 35.3 (SD 3.7), while males averaged 26.5 (SD 2.1) and 34.5 (SD 0.7), respectively. Gender did not significantly impact knowledge gains, with *P*=.79 for the pretest and *P*=.60 for posttest scores. Pediatricians had higher mean pretest scores 28.2 (SD 2.9) and posttest scores 37.4 (SD 2.4) than other health professionals 20.2 (SD 5.9) and 33 (SD 2.5), respectively. Specialty significantly affected pre- and posttest scores (*P*=.03 and *P*=.02, respectively). Health professionals with prior training attendance demonstrated a significant difference in pretest scores compared to those without prior attendance (*P*=.046).

Even though gender had little significant impact on test scores, knowledge improved across all educational modules.

When test scores were compared by specialty, pediatricians outperformed health care professionals in module 1 (defining, classifying, and determining malnutrition prevalence) and module 4 (managing pediatric malnutrition; *P*=.02 and *P*=.02, respectively). Details are presented in Table 3.

**Table 3.** Comparison of educational modules results by specialty.

Module and specialty		n	Pretest mean (SD)	Posttest mean (SD)	Paired *t* test (*df*; *P* value)		Mann-Whitney (*P* value)	
					Pretest	Posttest	Pretest	Posttest
**One**					−2.15 (8;.06)	−2.89 (8;.02)	−1.89 (.06)	−2.15 (.03)
	Health professionals	5	4.8 (1.6)	8.4 (0.5)				
	Pediatricians	5	6.6 (0.9)	9.4 (0.5)				
**Two**					−2.12 (8;.07)	−1.37 (8;.21)	−1.61 (.11)	−1.21 (.23)
	Health professionals	5	5.2 (2.8)	8.2 (0.8)				
	Pediatricians	5	8 (1)	9 (1)				
**Three**					−0.45 (8;.67)	−1.27 (8;.24)	−0.35 (.73)	−1.23 (.22)
	Health professionals	5	2.2 (0.8)	4.2 (0.4)				
	Pediatricians	5	2.4 (0.5)	4.6 (0.5)				
**Four**					−2.76 (8;.03)	−2.84 (8;.02)	−2.02 (.04)	−2.24 (.03)
	Health professionals	5	8 (1.9)	12.2 (1.5)				
	Pediatricians	5	11.2 (1.8)	14.4 (0.9)				

The effect size, calculated using Cohen *d*, was 2.25, indicating a very large effect, suggesting a substantial improvement in knowledge among participants from the pretest to the posttest. This finding highlights the significant impact of the online course on participants’ understanding of pediatric malnutrition.

The analysis of 30-item participant satisfaction questionnaire revealed a Cronbach α of .74, indicating a satisfactory level of internal consistency and reliability for the instrument.

Participants reported high satisfaction with the content presentation style (mean 3.16, SD 0.24), very high satisfaction with the scientific knowledge gained (mean 3.26, SD 0.30), and high satisfaction with the e-learning environment (mean 3.06, SD 0.4). The details can be accessed on Table S2 in Multimedia Appendix 1.

Most participants (6/10) preferred a combination of traditional and e-learning, while 3 preferred e-learning only, and 1 preferred traditional education.

## Discussion

### Principal Findings

This study investigates the efficacy of online learning in addressing pediatric malnutrition, a significant global health concern. The research identified 68 essential competencies for effective pediatric malnutrition management, encompassing knowledge, skills, and attitudes. These competencies were developed through a collaborative process with Syrian health care professionals, offering a valuable resource for future training initiatives. This study demonstrated that a self-directed online course significantly enhanced participants’ knowledge acquisition, highlighting the potential of e-learning as a scalable solution for addressing training needs in resource-constrained environments.

This study’s findings underscore the importance of comprehensive competency frameworks for addressing pediatric malnutrition, especially in challenging contexts such as Syria. The participants highly rated the online course, suggesting its effectiveness in bridging training gaps in resource-constrained settings. This study provides valuable insights for developing and implementing effective training initiatives to improve the management of pediatric malnutrition in resource-limited and conflict-affected settings.

This study expands upon existing research on essential competencies in medical education and the feasibility of e-learning in Syria [21-23]. While international organizations have focused on pediatric malnutrition in low-income countries [1-3], research specifically addressing the competencies required by pediatricians and other health care professionals to manage these cases has been limited. This study aimed to identify essential competencies for managing pediatric malnutrition and evaluating the effectiveness of e-learning modules in enhancing knowledge among health care professionals.

This study identified essential competencies by drawing upon existing training guides and WHO guidelines on SAM [24,25]. Although this study was conducted before the release of updated WHO guidelines in 2023 [26], future revisions of the identified competencies and online course content are recommended to align with these new recommendations.

This study builds upon previous work by Meeker et al [27] on a technical competency framework for nutrition in emergencies, specifically focusing on pediatric malnutrition and the needs of Syrian health professionals. While prior studies [11,27,28] have highlighted the importance of managing pediatric malnutrition, this research fills a gap by identifying the essential competencies pediatricians require to effectively manage these cases.

This study demonstrates the potential of e-learning to effectively scale up malnutrition management knowledge, a recognized challenge in most low- and middle-income countries [29,30]. Consistent with Annan et al’s [8] findings, knowledge acquisition was enhanced when the course was linked to career and academic progress. Participants reported increased learning and understanding of effective pediatric malnutrition management. These findings align with Choi et al’s [7] demonstration across 4 countries that e-learning enhanced health care practices and reduced malnutrition mortality through increased facility-based management of SAM. While this study’s time frame precluded measuring clinical outcomes, the significant knowledge gains could be attributed to the course’s practical relevance and scientifically sound content, as reflected in high participant approval [31].

Pediatricians’ pretest performance was positively influenced by previous malnutrition training, indicating knowledge retention [32]. This highlights the need for posttests and follow-up evaluations to compare the effectiveness of e-learning with traditional training, especially given its lower implementation costs per participant [8].

Developing the educational course in Arabic was a key challenge due to the limited availability of Arabic language online resources [33]. However, offering the course in the local language facilitated better knowledge comprehension and potentially increased long-term retention and practical application [34].

### Strength

This study highlights the potential of online learning to improve pediatric malnutrition management, especially in challenging environments such as Syria. By identifying essential competencies and demonstrating the effectiveness of a self-directed online course, it offers a model for developing similar training programs in other resource-constrained and conflict-affected areas. The course’s accessibility, adaptability, and positive feedback from participants suggest a promising way to address the shortage of skilled health care professionals in these regions.

### Limitation

This pilot study, conducted with a small sample size, limits the generalizability of the findings to a broader population of health care professionals. While this study demonstrates positive knowledge acquisition, it does not assess the impact on clinical practice or patient outcomes. Additionally, this study does not delve deeply into the specific challenges and facilitators of implementing the online course in the Syrian context, nor does it include long-term follow-up to assess the persistence of knowledge gains and their impact on clinical practice. To mitigate these limitations, we strived to recruit a diverse sample within our limited scope and used rigorous data collection and analysis methods.

### Future Research

Future research should expand on this study by (1) conducting larger studies with diverse participants to ensure findings are broadly applicable; (2) assessing the online course’s impact on real-world clinical practice and patient outcomes, not just knowledge acquisition; (3) understanding practical considerations and implementation strategies for e-learning in challenging environments such as Syria; (4) translating identified skills and attitudes into practical training methods; and (5) conducting long-term follow-up to track knowledge retention and its impact on clinical practice.

### Conclusion

This study demonstrates the effectiveness of self-directed online learning in improving knowledge and skills related to pediatric malnutrition management among Syrian health care professionals. This study identified 68 essential competencies across various domains, highlighting the breadth of knowledge needed for effective pediatric malnutrition management. The findings suggest e-learning as a powerful tool for scaling up training in challenging contexts such as Syria, while acknowledging the need for careful consideration of contextual factors.

## Supplementary material

10.2196/53151 Multimedia Appendix 1 The file contains three explanatory tables about qualitative interpretation of 5-point Likert scale measurements, questionnaire assessment results, and CHERRIES. CHERRIES: Checklist for Reporting Results of Internet E-Surveys.

## Abbreviations

SAM: severe acute malnutrition
SVU: Syrian Virtual University
UNICEF: United Nations Children’s Fund
WHO: World Health Organization
