# Supplementary material for: Evaluating the Effectiveness of an Online Course on Pediatric Malnutrition for Syrian Health Professionals: Qualitative Delphi Study
Source: JMIR Med Educ. 2024 Oct 28;10:e53151. doi: 10.2196/53151 (PMC11615703; doi:10.2196/53151)
Supplement: Multimedia Appendix 1 [file mededu-v10-e53151-s001.pdf]

Table S1. Qualitative Interpretation of 5-Point Likert Scale Measurements

Likert Scale Description	Likert Scale	Likert Scale Interval	Result Interpretation
Strongly disagree	0	0-0.79	Very low
Disagree	1	0.8-1.59	Low
Neutral/Uncertain	2	1.6-2.39	Medium
Agree	3	2.4-3.19	High
Strongly agree	4	3.2-4	Very high

Table S2. Questionnaire assessment results

Domain	Items (N)	Mean	SD*	Result interpretation
Content presentation style	10	3.16	0.24	High
Knowledge of scientific content	10	3.26	0.30	Very high
E-learning environment	10	3.06	0.4	High

*SD: standard deviation

Table S3. Checklist for Reporting Results of Internet E-Surveys (CHERRIES)

Checklist Item	*Explanation*
Describe survey design	Describe target population, sample frame. Is the sample a convenience sample? (In "open" surveys this is most likely.)<br>The target population are online course Participants who completed pre-and post-tests. This sample includes pediatricians and health professionals those who deal with malnutrition in children.
IRB approval	Mention whether the study has been approved by an IRB.<br>This study was approved by the Ethical Committee at the Syrian Virtual University (SVU) (#2154/0, November 25, 2021).
Informed consent	Describe the informed consent process. Where were the participants told the length of time of the survey, which data were stored and where and for how long, who the investigator was, and the purpose of the study?<br>The announcement of participation in the research was published via the researcher's e-mail and on social networking sites within the official groups for doctors and resident students for those who wish to participate in the electronic program (optional participation, not compulsory). The aim of the research was made clear that it is for scientific research only, and the average grades of the participants will not be used, except within this research and in a confidential manner, without mentioning personal data.<br>Participants were also explicitly asked about their consent via e-mail, and the response was either yes or no via e-mail as well.<br>The data was stored electronically on the Virtual University server or private e-mail.<br>The data was not anonymous or encrypted.<br>The stored data was subject to the security conditions of the Virtual University, and electronic access was determined for researchers according to their authority via username and password.<br>The data were not cleaned after being used in this research with the aim of anticipating their usefulness for subsequent research comparisons.<br>The information enterer can complete it in parts, but the electronic data collection system does not allow amending the data after entering it completely and approving the information that has been written, with the aim of avoiding tampering with the information after confirming it.<br>Overall data management is the responsibility of the researchers<br>The electronic virtual platform of the Syrian Virtual University was used.
Data protection	If any personal information was collected or stored, describe what mechanisms were used to protect unauthorized access.<br>The stored data was subject to the security conditions of the Virtual University, and electronic access was determined for researchers according to their authority via username and password.
Development and testing	State how the survey was developed, including whether the usability and technical functionality of the electronic questionnaire had been tested before fielding the questionnaire.<br>The survey includes 30 short and easy to understand items, which are assessing satisfaction with the content, organization, and learning environment of online course. A Likert scale ranging from 0 to 4 was utilized. A link of questionnaire was sent to participants via email
Open survey versus closed survey	An "open survey" is a survey open for each visitor of a site, while a closed survey is only open to a sample which the investigator knows (password-protected survey).<br>The survey was only open to the research sample (password-protected survey).
Contact mode	Indicate whether or not the initial contact with the potential participants was made on the Internet. (Investigators may also send out questionnaires by mail and allow

	for Web-based data entry.)<br>The initial contact with the potential participants was made on the Internet. Investigators also send out questionnaires by mail.
Advertising the survey	How/where was the survey announced or advertised? Some examples are offline media (newspapers), or online (mailing lists – If yes, which ones?) or banner ads (Where were these banner ads posted and what did they look like?). It is important to know the wording of the announcement as it will heavily influence who chooses to participate. Ideally the survey announcement should be published as an appendix.<br>The announcement of participation in the research was published via the researcher's e-mail and on social networking sites within the official groups for doctors and resident students for those who wish to participate in the electronic program (optional participation, not compulsory).
Web/E-mail	State the type of e-survey (eg, one posted on a Web site, or one sent out through e-mail). If it is an e-mail survey, were the responses entered manually into a database, or was there an automatic method for capturing responses?<br>It was an e-survey sent out through e-mail.<br>The responses were entered by an automatic method (Google Form).
Context	Describe the Web site (for mailing list/newsgroup) in which the survey was posted. What is the Web site about, who is visiting it, what are visitors normally looking for? Discuss to what degree the content of the Web site could pre-select the sample or influence the results. For example, a survey about vaccination on a anti-immunization Web site will have different results from a Web survey conducted on a government Web site<br>The announcement of participation in the research was published via the researcher's e-mail and on social networking sites within the official groups for doctors and resident students for those who wish to participate in the online course.
Mandatory/voluntary	Was it a mandatory survey to be filled in by every visitor who wanted to enter the Web site, or was it a voluntary survey?<br>It was a mandatory survey to be filled in by all participants who completed the pre-and post-test.
Incentives	Were any incentives offered (eg, monetary, prizes, or non-monetary incentives such as an offer to provide the survey results)?<br>No, the participation in the research was voluntary without any incentives.
Time/Date	In what timeframe were the data collected?<br>The results of the questionnaire were collected a week after it was sent to the participants who completed the tests.
Randomization of items or questionnaires	To prevent biases items can be randomized or alternated.<br>The distribution of items was random when sending the questionnaire to participants to prevent bias, and alternative items were also placed to ensure the validity and accuracy of the answers.
Adaptive questioning	Use adaptive questioning (certain items, or only conditionally displayed based on responses to other items) to reduce number and complexity of the questions.<br>Not used
Number of Items	What was the number of questionnaire items per page? The number of items is an important factor for the completion rate.<br>There were 10 items per page.
Number of screens (pages)	Over how many pages was the questionnaire distributed? The number of items is an important factor for the completion rate.<br>The questionnaire was distributed over 3 pages.
Completeness check	It is technically possible to do consistency or completeness checks before the questionnaire is submitted. Was this done, and if "yes", how (usually JAVAScript)? An alternative is to check for completeness after the questionnaire

	has been submitted (and highlight mandatory items). If this has been done, it should be reported. All items should provide a non-response option such as “not applicable” or “rather not say”, and selection of one response option should be enforced.<br>Questionnaire internal consistency was calculated with Cronbach's alpha, which was found to be 0.743.<br>For this 5-point Likert scale was used, and the range is 4 (highest value 5 minus lowest value 1). With 5 categories, the interval length is 4/5 = 0.80
Review step	State whether respondents were able to review and change their answers (eg, through a Back button or a Review step which displays a summary of the responses and asks the respondents if they are correct).<br>Yes, this was made available to participants through Google Form settings.
Unique site visitor	If you provide view rates or participation rates, you need to define how you determined a unique visitor. There are different techniques available, based on IP addresses or cookies or both.<br>There was no need for this, because all participants answered the questionnaire after the end of the educational course and within the required period.
View rate (Ratio of unique survey visitors/unique site visitors)	Requires counting unique visitors to the first page of the survey, divided by the number of unique site visitors (not page views!). It is not unusual to have view rates of less than 0.1 % if the survey is voluntary.<br>The participation in the research was voluntary, but the questionnaire was mandatory for all participants.
Participation rate (Ratio of unique visitors who agreed to participate/unique first survey page visitors)	Count the unique number of people who filled in the first survey page (or agreed to participate, for example by checking a checkbox), divided by visitors who visit the first page of the survey (or the informed consents page, if present). This can also be called “recruitment” rate.
Completion rate (Ratio of users who finished the survey/users who agreed to participate)	The number of people submitting the last questionnaire page, divided by the number of people who agreed to participate (or submitted the first survey page). This is only relevant if there is a separate “informed consent” page or if the survey goes over several pages. This is a measure for attrition. Note that “completion” can involve leaving questionnaire items blank. This is not a measure for how completely questionnaires were filled in. (If you need a measure for this, use the word “completeness rate”.)<br>The questionnaire completion rate was 100%.
Cookies used	Indicate whether cookies were used to assign a unique user identifier to each client computer. If so, mention the page on which the cookie was set and read, and how long the cookie was valid. Were duplicate entries avoided by preventing users access to the survey twice; or were duplicate database entries having the same user ID eliminated before analysis? In the latter case, which entries were kept for analysis (eg, the first entry or the most recent)?<br>The duplicate entries were avoided by preventing user’s access to the survey twice.
IP check	Indicate whether the IP address of the client computer was used to identify potential duplicate entries from the same user. If so, mention the period of time for which no two entries from the same IP address were allowed (eg, 24 hours). Were duplicate entries avoided by preventing users with the same IP address access to the survey twice; or were duplicate database entries having the same IP address within a given period of time eliminated before analysis? If the latter, which entries were kept for analysis (eg, the first entry or the most recent)?<br>The duplicate entries were avoided by preventing user’s access to the survey twice.
Log file analysis	Indicate whether other techniques to analyze the log file for identification of

	multiple entries were used. If so, please describe.<br>The other techniques to analyze the log file for identification of multiple entries were not used.
Registration	In “closed” (non-open) surveys, users need to login first and it is easier to prevent duplicate entries from the same user. Describe how this was done. For example, was the survey never displayed a second time once the user had filled it in, or was the username stored together with the survey results and later eliminated? If the latter, which entries were kept for analysis (eg, the first entry or the most recent)?<br>The username was stored together with the survey results and later eliminated, and the most recent entries were kept for analysis.
Handling of incomplete questionnaires	Were only completed questionnaires analyzed? Were questionnaires which terminated early (where, for example, users did not go through all questionnaire pages) also analyzed?<br>The completed questionnaires were only analyzed.
Questionnaires submitted with an atypical timestamp	Some investigators may measure the time people needed to fill in a questionnaire and exclude questionnaires that were submitted too soon. Specify the timeframe that was used as a cut-off point, and describe how this point was determined.<br>The questionnaire was allowed to be answered within a period of one week and the data analyzed after this period.
Statistical correction	Indicate whether any methods such as weighting of items or propensity scores have been used to adjust for the non-representative sample; if so, please describe the methods.<br>This method is not used.
